# An Improved IHBA-BP Neural Network for Temperature Compensation of Load Cells

**DOI:** 10.3390/s26123691

**Published:** 2026-06-10

**Authors:** Zhen-Jie Zhang, Wan-Sheng Cheng, Dai-Xing Zhang

**Affiliations:** 1 School of Electronic and Information Engineering, University of Science and Technology Liaoning, Anshan 114051, China; zzj512682701@126.com; 2 School of Mechanical Engineering and Automation, University of Science and Technology Liaoning, Anshan 114051, China; zdxing2005@126.com

**Keywords:** temperature compensation, load cell, IHBA-BP model, experimental calibration system

## Abstract

**Highlights:**

**What are the main findings?**

- The proposed IHBA integrates uniform initialization, a nonlinear weight factor, and Lévy flight with stagnation-aware triggering to overcome the limitations of the standard HBA.
- IHBA is applied for the first time to optimize a BP neural network for load cell temperature compensation over the 0–60 °C range, addressing both zero drift and sensitivity drift.

**What is the implication of the main finding?**

- Based on the experimental results, the IHBA-BP model reduces the zero-drift coefficient by 86.6% and the sensitivity-drift coefficient by 95.1%, outperforming four benchmark methods.

**Abstract:**

Temperature variations degrade load cell accuracy. To address this problem, an Improved Honey Badger Algorithm (IHBA) was developed to optimize the weights and biases of a BP neural network. IHBA incorporates uniform initialization, a nonlinear weight factor, and Lévy flight with stagnation-aware triggering to overcome the uneven initialization, poor exploration–exploitation balance, and weak local optima escape capability of the standard HBA. To validate the proposed method, a dedicated calibration experimental system was constructed. A 7075 T6 aluminum load cell (50 kN) was tested under 0–50 kN loading–unloading cycles over a temperature range of 0–60 °C. To eliminate random errors, three identical elastomers were fabricated, each tested three times, and the measured values were averaged. The results show that after IHBA-BP compensation, the zero-temperature drift coefficient of the load cell was reduced from 374.8 ppm/°C to 35.09 ppm/°C, and the sensitivity-temperature coefficient was reduced from 936.94 ppm/°C to 45.75 ppm/°C. On the unseen test set, the relative error after compensation was 0.01207, the mean square error was 2.84 × 10^−5^, and the root mean square error was 0.00533. Compared with IMA-BP, PSO-BP, BP, and polynomial fitting methods, IHBA-BP achieved the lowest error. The proposed method shows strong potential for industrial load cell temperature compensation.

## 1. Introduction

In aerospace, advanced manufacturing, and metallurgical industries [1,2,3], precise control of machining forces is the key to ensuring product quality, which relies on high-precision load cells. The measurement performance of conventional strain-gauge load cells is susceptible to various external environmental factors, including electromagnetic interference, temperature variations, and mechanical fatigue [4,5,6]. Among these factors, temperature variation has the most significant influence. The zero drift and sensitivity drift induced by temperature variations can severely degrade the measurement accuracy of the sensor [7]. To eliminate this measurement error, two temperature compensation methods are commonly used: hardware compensation and software compensation [8,9]. Hardware compensation methods include bridge circuit compensation, resistor compensation and diode compensation [10,11]. Nevertheless, hardware compensation suffers from high costs and poor flexibility. Particularly when compensating over a wide temperature range, it often fails to deliver satisfactory results [12].

Compared with the hardware approaches, software compensation offers lower cost and greater flexibility. Software compensation methods include curve interpolation, polynomial fitting [13], multi-dimensional regression [14] and the least squares support vector machine (LSSVM) algorithm [15,16]. However, these methods exhibit certain limitations in temperature compensation applications. Curve interpolation and polynomial fitting methods are prone to overfitting when applied to sensor temperature compensation. The multi-dimensional regression method imposes strict requirements on the accuracy of the predefined functional form. In addition, LSSVM and least squares surface fitting methods may require considerable computational resources during implementation. Owing to their capability for modeling complex nonlinear relationships, neural networks have been widely applied in sensor temperature compensation. Futane et al. [17] applied an Artificial Neural Network (ANN) algorithm to compensate for temperature drift in pressure sensors. The network learned the nonlinear mapping between temperature and sensor output from experimental data, adjusting weights and thresholds iteratively to reduce compensation error. Zhang et al. [18] employed a Back Propagation (BP) neural network for strain sensor compensation and found that the temperature-induced error was significantly reduced. Subsequently, Pang et al. [19] proposed a temperature compensation method based on a Radial Basis Function (RBF) neural network.

Neural networks can achieve high compensation accuracy. However, their performance depends heavily on the initialization of weights and thresholds, which may restrict their robustness and stability. To alleviate these limitations, researchers have introduced metaheuristic algorithms to optimize neural network parameters. Owing to its simplicity and effectiveness, Particle Swarm Optimization (PSO) [20] has been widely applied to neural network optimization. Gordan et al. [21] proposed a PSO-optimized ANN for sensor applications. They demonstrated that the convergence of the PSO-ANN was better than that of the ANN. Wu et al. [22] demonstrated that the PSO-RBF network achieved better temperature compensation performance than the unoptimized RBF model.

Nevertheless, PSO suffers from premature convergence and sensitivity to parameter settings, which may degrade convergence performance and compensation stability [23]. Consequently, increasing attention has been devoted to recently developed metaheuristic optimization strategies. Wang et al. [24] proposed a temperature compensation scheme for a multi-channel pressure scanner, in which an improved Cuckoo Search (CS) algorithm [25] was employed to optimize a BP neural network. Li et al. [26] applied the Black Widow Optimization (BWO) algorithm to optimize a BP neural network for fiber-optic sensor temperature compensation. Subsequently, Wang et al. [27] employed a genetic algorithm-optimized BP neural network to compensate for the temperature drift of a MEMS resonant accelerometer. Li et al. [28] utilized the ISSA-BP neural network to mitigate the influence of temperature drift on sensor output.

Moreover, deep learning methods such as Long Short-Term Memory (LSTM) [29] and hybrid deep learning models [30] have also been used for temperature compensation. Although these methods exhibit strong compensation capability, they typically require large amounts of data and substantial computational resources. As a result, their deployment in industrial applications remains limited. In contrast, neural network compensation methods based on metaheuristic algorithms are more lightweight. However, their performance heavily depends on the search capability of the selected algorithm. Therefore, choosing an appropriate metaheuristic algorithm plays a crucial role in temperature compensation.

The Honey Badger Algorithm (HBA) [31], proposed by Hashim et al. in 2022, is a recently developed metaheuristic inspired by the foraging behavior of honey badgers. It features a balance between exploration and exploitation, strong global search capability, and relatively few adjustable parameters. These characteristics make it a promising candidate for optimizing neural network-based temperature compensation models. However, the standard HBA still exhibits several inherent limitations. During the digging phase, the algorithm tends to overexploit the current best solution, leading to a rapid loss of population diversity and premature convergence. In later iterations, the honey phase excessively shrinks the search radius, thereby weakening the global exploration capability. Moreover, the algorithm demonstrates limited adaptability under varying conditions [32,33].

To solve these problems, various strategies have been introduced to improve HBA, such as hybrid differential evolution [34], Lévy flight [35], Tent mapping [36], and symbiotic mechanism-based approaches [37]. The above strategies have been proven effective in improving HBA performance for various optimization problems. However, most of these improvements focus on a single aspect (e.g., initialization, weight update, or local escape). They were evaluated on generic benchmark functions rather than on BP network optimization for load cell temperature compensation. Moreover, few studies simultaneously address three coupled issues: uneven initialization, imbalance between exploration and exploitation, and mid-iteration stagnation.

To overcome these limitations, this paper proposes an improved Honey Badger Algorithm (IHBA) specially designed for BP neural network optimization in load cell temperature compensation. The main novelties of this work are threefold:

- Algorithmic improvement: To overcome the limitations of the standard HBA, the proposed IHBA incorporates three strategies: uniform population initialization, a nonlinear weight factor in the digging phase, and a stagnation-aware triggering mechanism.
- Application scenario: IHBA is applied for the first time to optimize a BP neural network for load cell temperature compensation over the temperature range of 0–60 °C, addressing both zero drift and sensitivity drift.
- Experimental validation: Experimental results demonstrate that the IHBA-BP model reduces the zero-drift coefficient by 86.6% and the sensitivity-drift coefficient by 95.1%, outperforming four benchmark methods.

The structure of this paper is as follows. Section 2 analyzes the causes of temperature drift. Section 3 presents the temperature compensation method based on the BP neural network and IHBA. Section 4 introduces the experimental test system for the load cell. Subsequently, Section 5 analyzes the experimental results using the proposed temperature compensation method. Finally, Section 6 presents the conclusions.

## 2. Theoretical Analysis of Temperature Drift

For a strain gauge, the relationship between the resistance of a strain gauge and temperature can be expressed as

(1) $$R_{t}=R_{0}\left(1+\alpha_{0}\Delta t\right)$$

where *R_t_* and *R*_0_ represent the resistance values of the strain gauge at temperatures *t* and 0 degrees respectively. *α*_0_ represents the resistance temperature coefficient of the strain-gauge resistance wire. Δ*t* is the temperature difference. When the temperature changes by Δ*t*, the change in resistance of the strain gauge is given by Equation (2).

(2) $$\Delta R_{\alpha }=R_{t}-R_{0}=R_{0}\alpha_{0}\Delta t$$

In a strain-gauge load cell, the principle of thermal expansion causes changes in ambient temperature to produce synchronous dimensional changes in both the elastomer and the strain gauge. When the elastomer and the strain-gauge resistance wire have the same coefficient of thermal expansion, variations in ambient temperature do not produce additional deformation. However, due to the difference in their coefficients of thermal expansion, the elastomer and the strain gauge undergo unequal dimensional changes under the same temperature variation.

Assume that the lengths of the strain-gauge resistance wire and the elastomer are both *L*_0_ at 0 °C. Their coefficients of thermal expansion are denoted by *β_s_* and *β_g_*, respectively. If the strain gauge is not attached to the elastomer, the lengths of the resistance wire *L_s_* and the elastomer *L_g_* can be expressed as follows:

(3) $$\begin{array}{l} L_{s}=L_{0}\left(1+\beta_{s}\Delta t\right)\\ L_{g}=L_{0}\left(1+\beta_{g}\Delta t\right) \end{array}$$

If the strain gauge is attached to the elastomer, the additional deformation Δ*L*, the additional strain *ε_β_* and the additional resistance Δ*R_β_* of the strain-gauge resistance wire are shown as

(4) $$\begin{array}{l} \Delta L=L_{g}-L_{s}=\left(\beta_{g}-\beta_{s}\right)L_{0}\Delta t\\ \varepsilon_{\beta }=\frac{\Delta L}{L_{0}}=\left(\beta_{g}-\beta_{s}\right)\Delta t\\ \Delta R_{\beta }=KR_{0}\varepsilon_{\beta }=KR_{0}\left(\beta_{g}-\beta_{s}\right)\Delta t \end{array}$$

The relative change in the total resistance of the strain gauge caused by temperature is

(5) $$\frac{\Delta R_{t}}{R_{0}}=\frac{\Delta R_{\alpha }+\Delta R_{\beta }}{R_{0}}=\left[\alpha_{0}+K\left(\beta_{g}-\beta_{s}\right)\right]\Delta t$$

The strain caused by changes in ambient temperature is given as Equation (6). This phenomenon is called zero-temperature drift.

(6) $$\varepsilon_{t}=\frac{\Delta R_{t}/R_{0}}{K}=\left[\frac{\alpha_{0}}{K}+\left(\beta_{g}-\beta_{s}\right)\right]\Delta t$$

The zero-temperature coefficient *γ*_0_ is a parameter that quantifies how much the sensor’s zero-point drifts with temperature, as defined in Equation (7).

(7) $$\gamma_{0}=\frac{U_{T}-U_{T_{0}}}{U_{FS0}\left(T-T_{0}\right)}\times 10^{6}\;(\mathrm{ppm}/{}^{\circ}C)$$

where *U_T_* is the no-load output voltage at temperature *T*, *U_T_*_0_ is the no-load output voltage at temperature *T*_0_, and *U_FS_*_0_ is the full-load output voltage at *T*_0_.

The input–output characteristic of the load cell also varies with temperature, a phenomenon known as sensitivity-temperature drift.

The sensitivity-temperature coefficient *γ_s_* quantifies the change in the sensor’s sensitivity with temperature, which is given by

(8) $$\gamma_{s}=\frac{S_{T}-S_{T_{0}}}{S_{T_{0}}\left(T-T_{0}\right)}\times 10^{6}\;(\mathrm{ppm}/{}^{\circ}C)$$

where *S_T_* is the sensitivity measured at the specific temperature under *T*, and *S_T_*_0_ is the sensitivity at the reference temperature *T*_0_.

**Figure 1:**
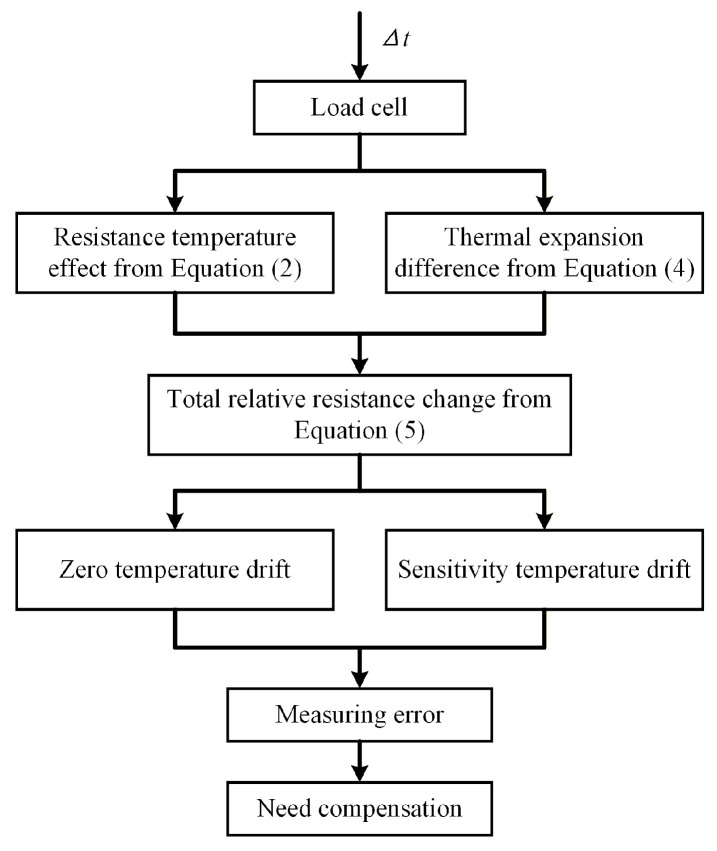
Flowchart of the temperature drift mechanism in a load cell.

Zero-temperature drift and sensitivity-temperature drift collectively constitute temperature drift [38]. From Equations (5)–(8), it is evident that both zero-temperature drift and sensitivity-temperature drift depend on the temperature variation Δ*t* and the material parameters. Moreover, zero drift occurs even under no-load conditions, whereas sensitivity drift changes the force–output scaling relationship. These two effects are not independent; both vary nonlinearly with temperature and applied load. Therefore, a simple linear correction cannot completely eliminate temperature-induced errors over a wide temperature range, and a more sophisticated nonlinear model is required. The flowchart illustrating the temperature drift mechanism of the load cell is shown in Figure 1.

## 3. Methodology

**Figure 2:**
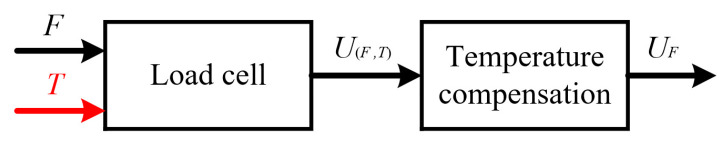
The principle of a load cell temperature compensation.

The principle of temperature compensation for a load cell is shown in Figure 2. *F* represents the measured force, and *T* represents the measured ambient temperature. Under the combined influence of force and temperature, the output signal of the load cell is a mixed signal, *U*_(*F*, *T*)_, which contains both the force-related signal and the temperature-induced disturbance. The goal of temperature compensation is to establish a mapping model capable of recovering the force signal *U_F_* from the mixed signal *U*_(*F*, *T*)_. Because this mapping relationship is highly nonlinear, simple linear correction methods cannot achieve the required accuracy. Therefore, this paper employs a BP neural network to learn this nonlinear mapping relationship, and the network parameters are optimized using the proposed IHBA.

### 3.1. BP Neural Network

The BP neural network is a multilayer feedforward neural network based on the error backpropagation algorithm. BP neural networks are widely used in classification, regression, pattern recognition, and other fields. Their core principle is to adjust the weights and bias parameters, thereby enabling the network to approximate complex nonlinear functions. Owing to its strong nonlinear approximation capability, the BP neural network is selected as the core algorithm for load cell temperature compensation.

**Figure 3:**
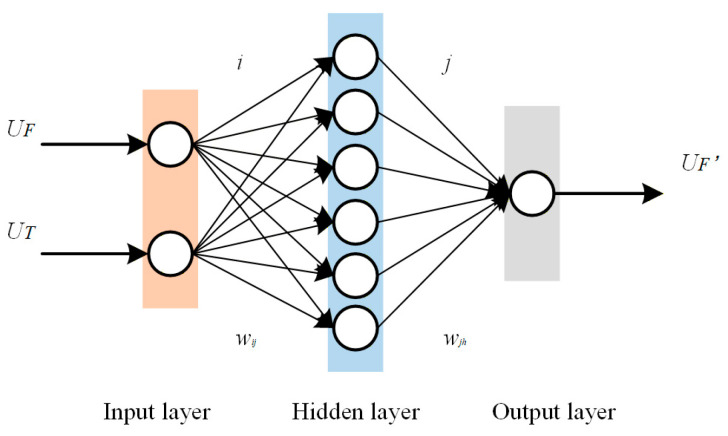
Basic structure of a practical 3-layer BP neural network.

Figure 3 illustrates the basic structure of a practical three-layer BP neural network. It consists of three parts: the input layer, the hidden layer, and the output layer. Each node represents a neuron. The working mechanism of the BP neural network is mainly divided into two stages: forward propagation and backward propagation.

Forward propagation: The input signals are processed through weighted summation and then transmitted to the neurons in the next layer through an activation function, ultimately generating the output result, as shown in Equation (9). In Equation (9), *w_ij_* represents the weights, and *b_j_* denotes the bias. If the error between the output result and the calibrated result exceeds a predefined threshold, the process proceeds to the backward propagation stage. The error between the output result and the calibrated result is calculated using Equation (10). To evaluate the effectiveness of the compensation, *U_F_*′ is defined as the output result, while *U_Fn_* is defined as the calibrated result.

(9) $$U_{j}=\sum_{i}\omega_{ij}U_{i}+b_{j}$$

(10) $$E=\frac{1}{2}{\left[U_{F}-U_{Fn}\right]}^{2}$$

Backward propagation: The error at the output layer is calculated and propagated backward through the network to the hidden layer. The connection weights of each layer are then updated using the gradient descent method to minimize the overall error.

The initial weights and biases of the BP neural network are typically assigned randomly, which may result in an uneven distribution of candidate solutions within the search space. Consequently, the network may converge prematurely without sufficiently exploring the global optimum region. To address this limitation, a metaheuristic algorithm with strong exploration capability, namely the Honey Badger Algorithm (HBA), is introduced to optimize the BP neural network. The initial weights and biases are determined through the HBA search process, thereby enhancing the global search capability and improving optimization efficiency.

### 3.2. Mathematical Model of HBA

The HBA is inspired by the foraging behavior of honey badgers, which includes two strategies: digging and following honeyguide birds. These behaviors correspond to the digging mode and honey mode of the algorithm, respectively. In the digging mode, the honey badger uses its sense of smell to locate prey and then moves around the target area to select an appropriate digging position. In the honey mode, the honey badger directly locates prey by following a honeyguide bird. The detailed steps of the HBA are as follows:

Step 1: Initialization phase. Initialize the position of each honey badger based on Equation (11).

(11) $$x_{i}=lb_{i}+r_{1}\times \left(ub_{i}-lb_{i}\right)$$

where *x_i_* represents the initial position of the ith honey badger, *lb_i_* is the lower bound, *ub_i_* is the upper bound, *r*_1_ is a random number in the range (0 < *r*_1_ < 1), and *i* represents dimensionality.

Step 2. Defining intensity (I).

Intensity is related to the concentration of prey and the distance between the prey and the honey badger. The more concentrated the prey and the closer the distance between the honey badger and the prey, the greater the I, as expressed by Equation (12).

(12) $$I_{i}=r_{2}\times \frac{S}{4\pi d_{i}^{2}}$$

(13) $$S={\left(x_{i}-x_{i+1}\right)}^{2}$$

(14) $$d_{i}=x_{prey}-x_{i}$$

where *d_i_* is the distance between the prey and the ith badger, *r*_2_ is a random number between 0 and 1, and *S* denotes source strength or concentration strength.

Step 3. Update density factor.

The density factor α is given by Equation (15). It is used to balance exploration and exploitation.

(15) $$\alpha =C\times \exp\left(\frac{-t}{t_{\max}}\right)$$

where *C* is a constant, which is greater than 1; *t* is the current number of iterations; and *t_max_* is the maximum number of iterations.

Step 4. Update position.

In the previous discussion, HBA is divided into the digging phase and the honey phase.

#### 3.2.1. Digging Phase

The motion of the digging phase is calculated by

(16) $$x_{new}=x_{prey}+G\times \beta \times I\times x_{prey}+G\times r_{3}\times \alpha \times d_{i}\times \left[\cos\left(2\pi r_{4}\right)\times \left[1-\cos(2\pi r_{5})\right]\right]$$

where *x_prey_* is the global optimal point. *G* is used as a flag that alters the movement direction of the honey badger, and it is determined in Equation (17). *β* represents the ability to search for food (*β* ≥ 1). *r*_3_, *r*_4_, and *r*_5_ are three random numbers between 0 and 1.

(17) $$G=\left\{\begin{array}{c} 1\\ -1 \end{array}\begin{array}{c} if,r_{6}\leq 0.5\\ else \end{array}\right.$$

In Equation (17), *r*_6_ is a random number between 0 and 1. The value of *G* can be determined as follows: when *r*_6_ is less than 0.5, the value of *G* is 1; otherwise, the value of *G* is −1. This design ensures that *G* takes the values 1 and −1 with equal probability. Therefore, the honey badger can move in both positive and negative directions with equal likelihood, preventing directional bias that could lead to uneven coverage of the search space.

#### 3.2.2. Honey Phase

The motion of a honey badger following a honeyguide bird to a beehive is described by

(18) $$x_{new}=x_{prey}+G\times r_{7}\times \alpha \times d_{i}$$

where *r*_7_ is a random number between 0 and 1, and *x_prey_* represents the best candidate solution found thus far, which may be a local optimum rather than the global one. Employing a perturbation factor *r*_7_ is essential to facilitate escape from local optima and enhance the exploration toward the global best position.

### 3.3. Improvement of HBA

Despite its effectiveness, the standard HBA still suffers from three main limitations:

1. Uneven initial population distribution—Random initialization may lead to poorly distributed individuals, reducing the search efficiency at the early stage.
2. Imbalance between exploration and exploitation—During the digging phase, the algorithm tends to overexploit the current best solution, which rapidly decreases population diversity and may lead to premature convergence. In the honey phase, the search radius shrinks excessively in later iterations, weakening the global exploration capability.
3. Lack of an effective escape mechanism—The algorithm can easily become trapped in local optima during the middle and later stages of iteration and exhibits limited adaptability under varying conditions.

To overcome these limitations, several improvements are introduced into the HBA.

1. To improve the initial population distribution, MATLAB 2017’s unifrnd function is employed to initialize the population of HBA. This strategy produces a more uniformly distributed initial population and improves the search efficiency in the early stage.
2. To better balance between exploration and exploitation, a nonlinear weight factor *g* is considered in the digging phase, as described in Equations (19) and (20). The value of *g* decreases nonlinearly from 0.9 to 0.2 during the iteration process. A relatively large *g* in the early stage encourages global exploration, whereas a smaller *g* in later iterations enables the algorithm to perform more refined local searches and reduces unnecessary disturbances near the optimal solution.

(19) $$x_{new}=x_{prey}+g\times G\times \beta \times I\times x_{prey}+G\times r_{3}\times \alpha \times d_{i}\times \left[\cos\left(2\pi r_{4}\right)\times \left[1-\cos(2\pi r_{5})\right]\right]$$

(20) $$g=g_{\max}-\left(g_{\max}-g_{\min}\right)\exp(-5\frac{1}{t})$$

**Figure 4:**
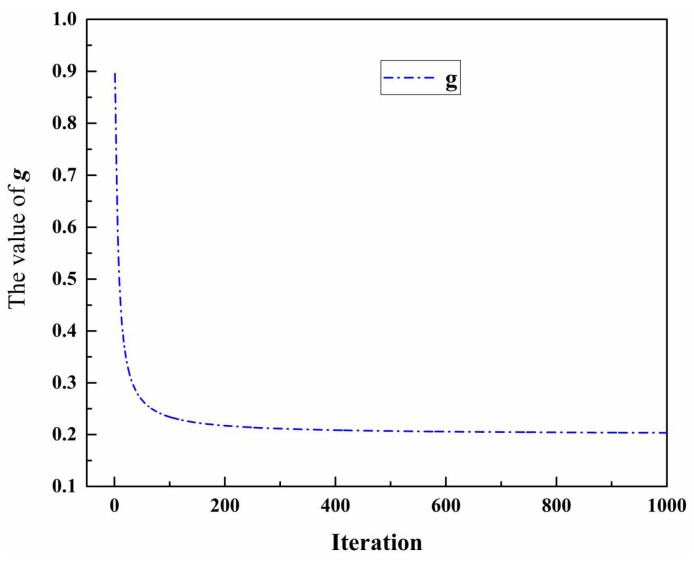
Variation in *g* with iteration number.

To illustrate the regulatory effect of the weight factor *g* on the exploration and exploitation capabilities of the algorithm during the iteration process, Figure 4 presents the variation curve of *g* with respect to the iteration number. At the early stage, *g* is close to its maximum value of 0.9, allowing the algorithm to move with large step sizes and thereby facilitating extensive global exploration. As the iteration proceeds, *g* decays nonlinearly, and the algorithm gradually shifts from global exploration to local exploitation. In the later stage, *g* approaches its minimum value of 0.2. The smaller search step size is beneficial for refined local search. This nonlinear decay strategy enables the algorithm to automatically balance exploration and exploitation at different stages of the optimization process, thereby avoiding premature convergence or slow convergence caused by a fixed weight factor.

**Figure 5:**
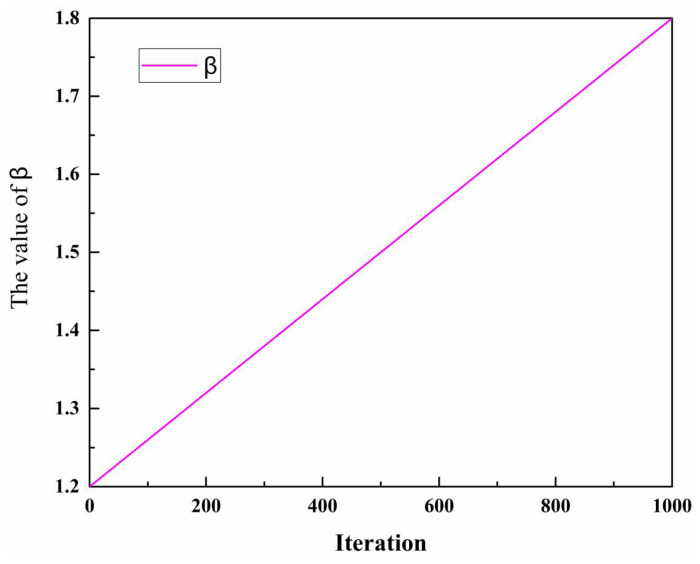
Variation in *β* with iteration number.

3. To improve the ability of the algorithm to escape local optima, a Lévy flight strategy is introduced into the honey phase [39], as described in Equation (21). Owing to its occasional long-distance jumps, Lévy flight can significantly enhance global exploration and effectively help the algorithm escape local optima. Meanwhile, the characteristic exponent *β* of the Lévy flight is adaptively adjusted. At the beginning of the iteration process, *β* is set to a relatively small value (*β* = 1.2), which produces large step sizes and enhances global exploration. As the iteration proceeds, *β* gradually increases to a larger value (*β* = 1.8), thereby reducing the step size and facilitating refined local search. This adaptive design further strengthens the algorithm’s capability to escape local optima. The variation curve of *β* with respect to the iteration number is shown in Figure 5. The new position of the honey badger can then be redefined using Equation (22).
  (21) $$Levy=\frac{u\times \sigma }{{\left|\nu \right|}^{-\beta }}$$
  (22) $$x_{new}=x_{prey}+L\mathrm{evy}\times F\times \alpha \times d_{i}$$
4. To further enhance global search capability and reduce the risk of premature convergence during the mid-to-late stages, the triggering probability of the honey phase is increased. Specifically, when convergence stagnation is detected during the iteration process (e.g., the best solution remains unchanged for 5 or 10 consecutive iterations), the triggering probability is increased to encourage the algorithm to explore a wider search region.

The above improvements are designed to achieve a better balance between global exploration and local exploitation. The nonlinear weight factor mainly improves exploration capability during the early stage, while the Lévy flight strategy improves the probability of escaping local optima. The adaptive triggering mechanism further stabilizes convergence during the later stage. These improvements jointly contribute to a more robust optimization process.

### 3.4. IHBA-BP Model

**Figure 6:**
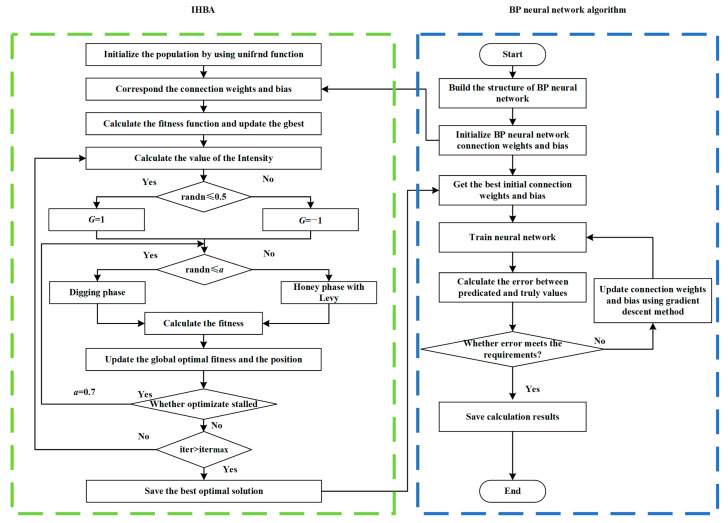
Flowchart of the IHBA-BP model for load cell temperature compensation.

Temperature compensation for the load cell is carried out using the IHBA-BP model. The optimal weights and biases obtained by IHBA are used as the initial weights and biases of the BP neural network, which can improve convergence accuracy and shorten search time. To further enhance compensation accuracy, the gradient descent method is applied in the IHBA-BP model. The flowchart of the proposed model is shown in Figure 6.

## 4. Calibration Experimental System

To investigate the output characteristics of the load cell under different temperatures and to perform thermal compensation, an experimental calibration system was built. The system consisted of an experimental test system, a load cell module and a strain-gauge adhesive device.

### 4.1. Experimental Test System

**Figure 7:**
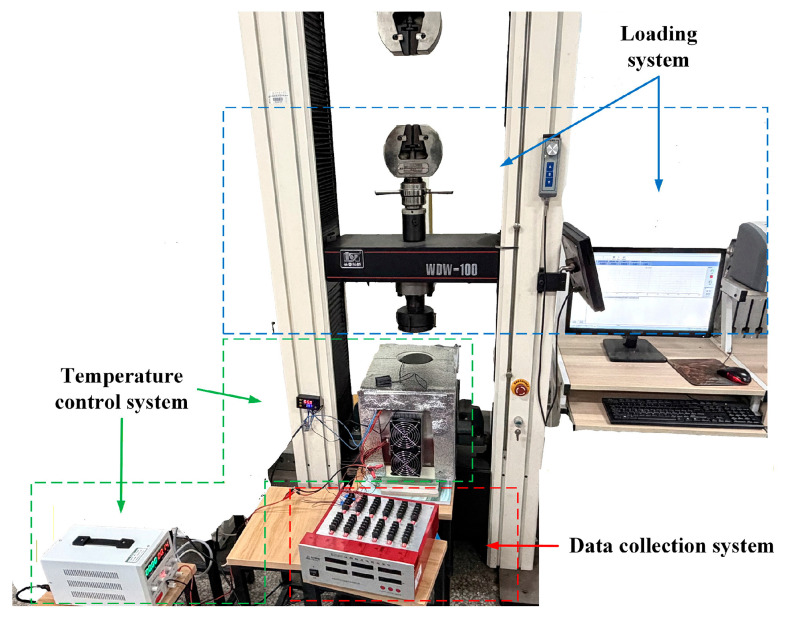
Temperature compensation calibration system for load cells.

The experimental test system mainly included a temperature control system, a data acquisition system, and a loading system, as shown in Figure 7.

**Figure 8:**
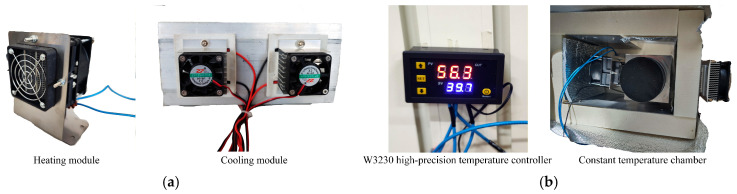
Temperature control system. (**a**) Heating and Cooling module (PTC heating and semiconductor cooling); (**b**) Constant-temperature chamber.

A temperature control system capable of real-time regulation was designed to measure the output signals of the elastomer under different temperature conditions. The system consisted of a heating module, a cooling module, a temperature control module, and a constant-temperature chamber. The heating module employed a PTC heating element (Wenzhou Youzhi Electric Co., Ltd., Wenzhou, China), while the cooling module utilized a semiconductor refrigeration component (Jinhua Tuglisi Technology Co., Ltd., Jinhua, China), as shown in Figure 8a. To achieve accurate temperature regulation, a W3230 high-precision temperature controller (Wenzhou Youzhi Electric Co., Ltd., Wenzhou, China) was adopted. All modules were installed inside the constant-temperature chamber, which was constructed using polyurethane insulation material and externally wrapped with heat-reflective aluminum foil to minimize heat exchange with the ambient environment, as shown in Figure 8b.

**Figure 9:**
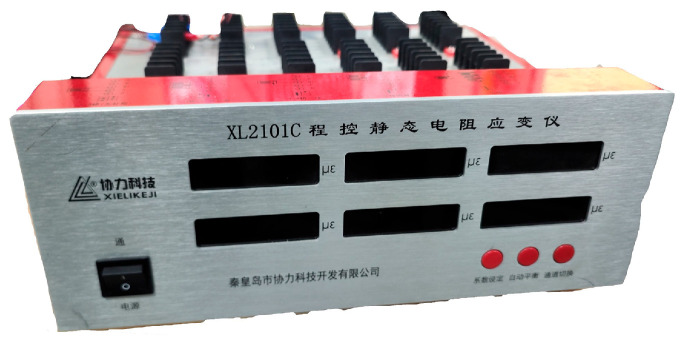
XL2101C static strain gauge used for data acquisition.

The load cell measurement circuit was configured as a full-bridge circuit, and the tests were conducted using an XL2101C static strain gauge (Qinhuangdao Xielì Technology Development Co., Ltd., Qinhuangdao, China). The XL2101C static strain gauge is shown in Figure 9. The measurement range of the instrument is −19,999 to +38,000 με, and the resolution is 1 με. The bridge excitation voltage is 2 V.

**Figure 10:**
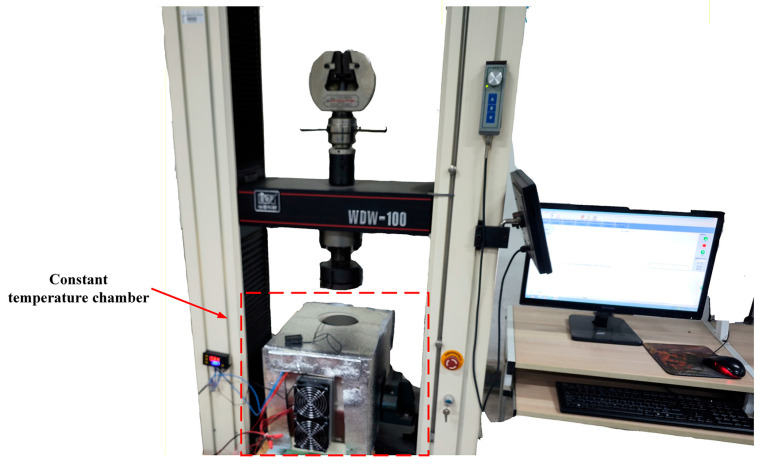
WDW-100 electronic universal testing machine for load application.

The loading system employed a calibrated 0.5-grade hydraulic universal testing machine, WDW-100 (Changchun Kexin Test Instrument Co., Ltd., Changchun, China), as the standard force source. The device is shown in Figure 10. This device has a maximum load capacity of 100 kN. WDW-100 has an accuracy class of 0.5, meaning its indication error is within ±0.5% of the full scale. This level of accuracy represents a common configuration in many industrial settings and typical laboratory environments.

### 4.2. Load Cell Elastomer

**Figure 11:**
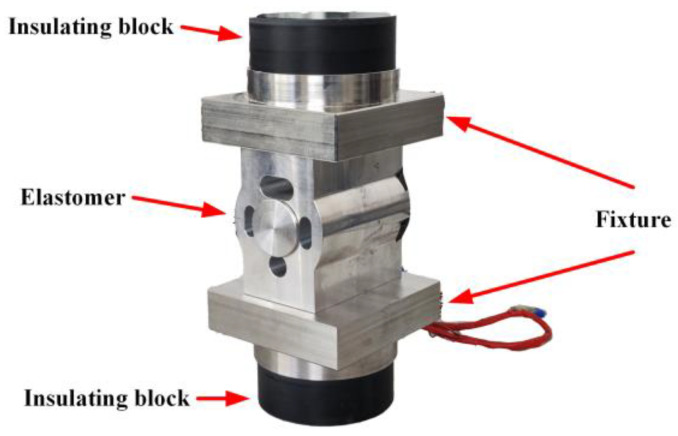
Load cell elastomer with a fixing device.

The elastomer was secured using a dedicated fixture to prevent movement, as shown in Figure 11. To reduce the influence of heat transfer from the loading head, a force-resistant ceramic insulation block was installed between the press head and the fixture, thereby effectively minimizing thermal conduction. The elastomer was fabricated from 7075-T6 aluminum alloy (Kunshan Shanji Metal Materials Business Department, Kunshan, China), which has an elastic modulus of 71 GPa and a yield strength of 445 MPa. To reduce random errors, three identical elastomers were manufactured, and the reported experimental results represent the average values obtained from all three specimens.

### 4.3. Strain-Gauge Adhesive Device

**Figure 12:**
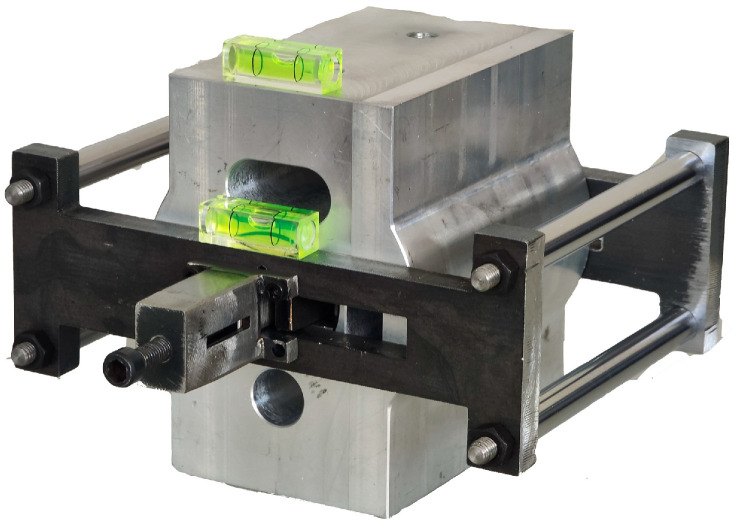
Strain-gauge adhesive device with linkage mechanism.

To enable strain-gauge (Zhonghang Electronic Measuring Instruments Co., Ltd., Hanzhong, China) bonding inside the central hole of the elastomer, a dedicated strain-gauge bonding device was designed in this study, as shown in Figure 12. Based on a linkage mechanism, the device converts the axial motion of the central shaft into radial movement of the strain gauge. This mechanism allows the strain gauge to be uniformly bonded to the inner wall of the elastomer’s central hole.

### 4.4. Calibration Methodology

#### 4.4.1. Experimental Setup and Parameters

The goal of the calibration experiment is to obtain the output characteristics of the load cell under different temperature and load conditions. The experimental setup is as follows:

1. Temperature control system: A W3230 high-precision temperature controller with an accuracy of ±0.1 °C was used.
2. Loading system: A WDW-100 electronic universal testing machine with a range of 0–100 kN and an accuracy class of 0.5 was employed.
3. Data acquisition: An XL2101C static strain gauge was configured as a full-bridge circuit, with a measurement range of −19,999 to +38,000 με, a resolution of 1 με, and a bridge excitation voltage of 2 V.
4. Load cell elastomer: The load cell was a plate-ring type made of 7075 T6 aluminum alloy with a rated capacity of 50 kN. Three identical elastomers were fabricated to reduce random errors.

#### 4.4.2. Calibration Procedure

1. Zero calibration: For load cell calibration, the output value at 20 °C is generally used as the reference condition. Therefore, before the experiment, the static strain gauge instrument was zero-calibrated under no-load conditions at 20 °C.
2. Temperature control: During the training stage, the temperature inside the constant-temperature chamber was increased from 0 °C to 60 °C in increments of 5 °C, resulting in 13 temperature points (0, 5, 10, …, 60 °C). At each target temperature, the system was maintained for 30 min to ensure thermal equilibrium. During the testing stage, the chamber temperature was increased from 27 °C to 57 °C in increments of 10 °C, resulting in four temperature points (27, 37, 47, and 57 °C). The system was similarly maintained for 30 min at each temperature point to achieve thermal equilibrium. Only the heating process was considered in this study, while temperature hysteresis during the cooling process was not investigated.
3. Load application and unloading: At each stabilized temperature, loads of 0, 10, 20, 30, 40, and 50 kN were sequentially applied. Each load was maintained for 5 s to allow the strain gauge output to stabilize before recording. After the loading sequence was completed, unloading was performed stepwise from 50 kN back to 0 kN in reverse order to evaluate load hysteresis, and the corresponding output values were recorded at each loading level.
4. Repeatability test: Three repeated tests were performed on each of the three fabricated elastomers. The final result was the average of these nine measurements.

#### 4.4.3. Evaluation Metrics

To comprehensively evaluate the performance of the subsequent temperature compensation algorithm, the relative error *e_r_*, full-scale error *e_f_*, mean square error (MSE), and root mean square error (RMSE) are introduced. Their definitions are provided in Equation (23).

(23) $$\left\{\begin{array}{l} e_{r}=\max\left|U_{r}-{\hat{U}}_{r}\right|,r=1,2,\ldots ,N\\ e_{f}=\max\left(\frac{\left|U_{r}-{\hat{U}}_{r}\right|}{U_{FS}}\right)\times 100\%,r=1,2,\ldots ,N\\ MSE=\frac{1}{N}{\sum_{i=1}^{N}\left(U_{r}-{\hat{U}}_{r}\right)}^{2},r=1,2,\ldots ,N\\ RMSE=\sqrt{\frac{1}{N}{\sum_{i=1}^{N}\left(U_{r}-{\hat{U}}_{r}\right)}^{2}},r=1,2,\ldots ,N \end{array}\right.$$

where *U_r_* is the measured voltage, $\hat{Ur}$ is the calibrated voltage under the reference condition (20 °C), *U_FS_* is the voltage corresponding to the full-scale output, and *N* is the number of measurement points.

## 5. Results and Discussion

### 5.1. Training Results

Based on the calibration experimental system and methodology, the training set presented in Table 1 consists of the averaged values.

### 5.2. Parameter Settings

The IHBA optimizer used a population size of 100 and a maximum iteration count of 1000, and the parameters were set to *β* = 6, *C* = 2.

The number of hidden layer neurons significantly affects the fitting and generalization performance of a BP neural network. To determine the optimal number, only the number of hidden layer neurons was varied. The number was varied from 2 to 10. For each candidate number, the model was run 10 times independently, and the results were averaged. The evaluation metrics on the training set included *e_r_*, *e_f_*, MSE, and RMSE. The simulation results are shown in Table 2.

**Figure 13:**
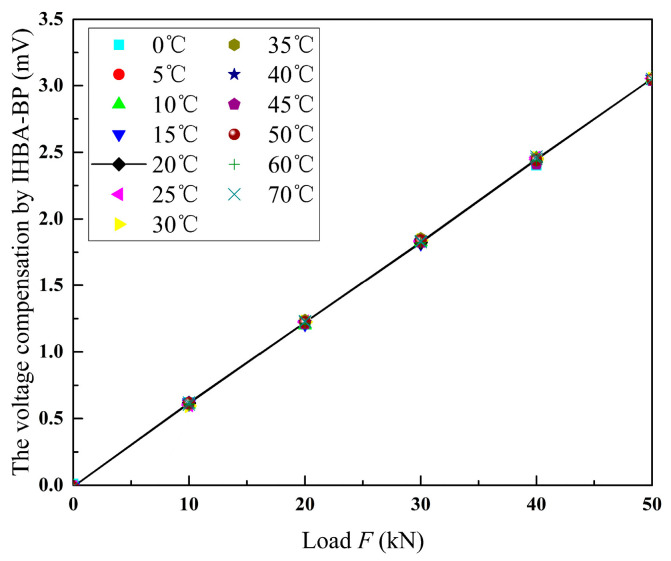
The compensation results from the IHBA-BP model.

The results showed that six neurons yielded the minimum values for all evaluation metrics, confirming that six was the optimal number. Moreover, the hyperbolic sine function was employed as the activation function between the input layer and the hidden layer. Load cell output is normalized within a bounded range. To better capture the nonlinear mapping characteristics and ensure stable output within the valid range, the Sigmoid activation function is adopted in the output layer. The compensation results are illustrated in Figure 13.

### 5.3. Dynamic Mechanism Analysis of IHBA

To examine how the IHBA algorithm enhances global exploration capability and mitigates premature convergence, this section analyzes the dynamic behavior of the algorithm during the training of the BP neural network.

#### 5.3.1. Analysis of the Algorithm Convergence

**Figure 14:**
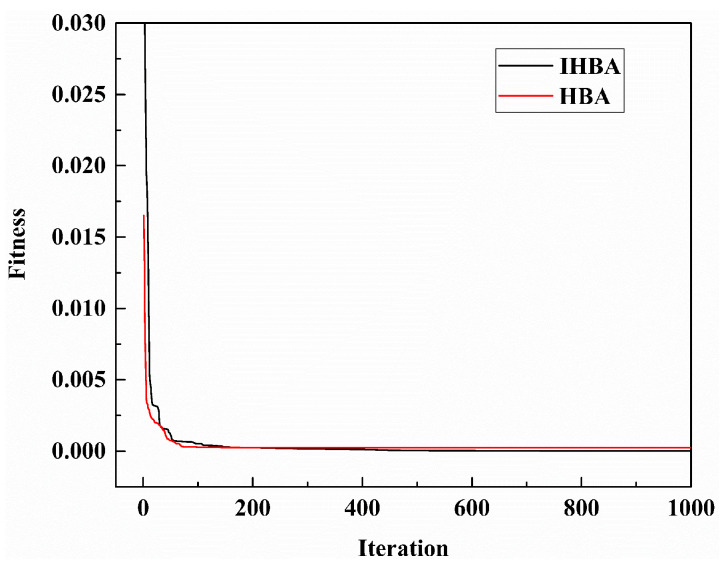
Convergence curves of the fitness.

Figure 14 shows the convergence curves of the fitness (training set MSE) for the standard HBA and the proposed IHBA. At the early stage of iterations, HBA has lower fitness than IHBA. However, IHBA exhibits several distinct stepwise drops between the 50th and 100th iterations. After these drops, IHBA maintains lower fitness than HBA until the end of the iterations. These stepwise drops correspond to the activation of the stagnation-aware mechanism. When no improvement is detected for ten consecutive iterations, the honey phase probability is increased and Lévy flight is triggered, enabling the algorithm to escape local optima. Although IHBA starts with a higher fitness, it achieves lower fitness than standard HBA after the 100th iteration. Thus, the proposed improvements effectively enhance global search capability and mitigate premature convergence.

#### 5.3.2. Analysis of Population Diversity Evolution

**Figure 15:**
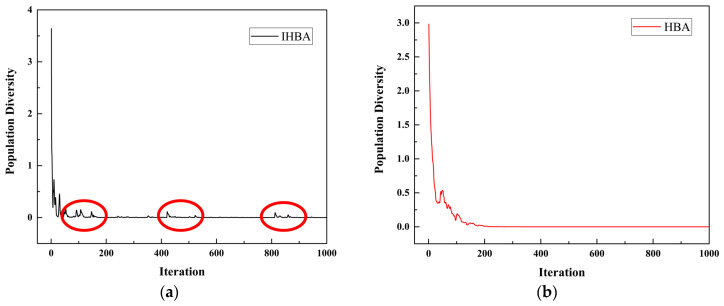
Diversity evolution curves: (**a**) Diversity evolution curves of IHBA; (**b**) Diversity evolution curves of HBA. Circles mark the step points where the stagnation-aware mechanism is activated.

To further analyze the global exploration capability and premature convergence mitigation of IHBA, the standard deviation of individuals across each dimension at each iteration was calculated. The average of these standard deviations over all dimensions was then used as the measure of population diversity. Figure 15 shows the diversity evolution curves of IHBA and HBA. In Figure 15a, the diversity of IHBA decreases rapidly at the early stage, indicating that the population quickly gathers around promising regions. Several step points appear in the curve, corresponding to the activation moments of the stagnation-aware mechanism. When no improvement is detected for ten consecutive iterations, the algorithm increases the honey phase probability and introduces Lévy flight. This enables the population to escape the current local optimum and continue moving toward a better solution. Figure 15b shows the HBA diversity curve. The average dimensional standard deviation of the HBA remains almost unchanged after the 200th iteration. This indicates that HBA only searches around the current best value and no longer produces significant changes, exhibiting typical premature convergence characteristics.

### 5.4. Comparison of Compensation Methods

The compensation results of IHBA-BP were compared with those obtained from four established compensation methods: a Particle Swarm Optimized BP neural network (PSO-BP), an improved Mayfly algorithm-optimized BP neural network (IMA-BP) [40], a standard BP neural network (BP), and a polynomial fitting method. To further evaluate the effectiveness of each improvement in IHBA, an ablation study was conducted by comparing the proposed method with its simplified variants.

The compensation results obtained using different methods were presented in Table 3. Compared with the raw, uncompensated data, the temperature drift coefficient of the sensor was reduced by 86.6% after IHBA-BP compensation, and the sensitivity-temperature coefficient was reduced by 95.86%, both of which meet the design specifications. In comparison with typical existing compensation methods, the proposed IHBA-BP model demonstrates significant advantages in terms of temperature drift coefficient, sensitivity-temperature coefficient, and various error metrics. The results also showed that each modification contributed to performance improvement, and the complete IHBA achieved the best results.

### 5.5. Test Results After Compensation

The proposed compensation model was applied to the unseen test set to further validate its effectiveness. Table 4 lists the samples in the test set, and Table 5 presents the corresponding compensation results.

Substituting the compensation results in Table 5 into Equation (23) yields the following error metrics: *e_r_* = 0.01207, MSE = 2.8376 × 10^−5^, and RMSE = 0.005327. Compared with the uncompensated case, the full-scale error *e_f_* was reduced by 78.6%, indicating that the proposed IHBA-BP model effectively suppresses temperature-induced errors.

Generalization analysis: The test set consists of four temperature points (27 °C, 37 °C, 47 °C, 57 °C) that were not used in training, and these points lie within the training temperature range (0–60 °C). The compensation errors on the test set (*e_r_* = 0.01207, MSE = 2.84 × 10^−5^) are close to those on the training set (*e_r_* = 0.0166, MSE = 2.66 × 10^−5^), with no significant increase, suggesting that the model does not overfit. Moreover, the compensation results are consistent across different load levels (0–50 kN, Table 5), with no load-dependent error fluctuations. Thus, the IHBA-BP model generalizes well to unseen temperatures and loads, making it suitable for industrial applications over a wide temperature range.

In summary, the proposed model achieves an accuracy level close to the instrument’s full-scale specification and maintains effective temperature compensation on the test set, showing promise for engineering applications.

## 6. Conclusions

This paper proposes a temperature compensation method for load cells based on a BP neural network optimized by an Improved Honey Badger Algorithm (IHBA). The standard HBA has three main limitations: uneven initial population distribution, poor balance between exploration and exploitation, and weak escape capability in mid-to-late iterations. To address these, the proposed IHBA introduces three targeted improvements: uniform initialization, a nonlinear weight factor in the digging phase, and a Lévy flight with a stagnation-aware triggering mechanism.

Experimental validation over a wide temperature range of 0–60 °C demonstrated the following main achievements of the proposed IHBA-BP model:

Significant reduction in temperature drift: The zero-drift coefficient decreased from 374.8 ppm/°C to 35.09 ppm/°C (86.6% reduction), and the sensitivity-drift coefficient decreased from 936.94 ppm/°C to 45.75 ppm/°C (95.1% reduction).

Superiority over existing methods: Compared with IMA-BP, PSO-BP, standard BP, and polynomial fitting, the IHBA-BP model achieved the lowest values across all evaluation metrics, including relative error, mean squared error, root mean squared error, and full-scale error.

Good generalization capability: On an unseen test set (27, 37, 47, 57 °C), the model reduced the full-scale error by 78.6%. The test errors were close to the training errors, indicating no overfitting and good generalization to unseen thermal conditions.

However, this study was validated only on a plate-ring load cell made of 7075 T6 aluminum alloy with a rated capacity of 50 kN, and only the heating process (0→60 °C) was considered. Temperature hysteresis (cooling cycle) was not evaluated. Because IHBA-BP is a data-driven method, its algorithmic framework does not depend on specific material or range parameters; therefore, it is transferable to other elastomer materials (e.g., stainless steel, beryllium bronze) and different load ranges once new calibration data are collected. Future work will (1) extend the validation to multiple sensor types and materials, and (2) introduce heating-cooling cycles to comprehensively evaluate and compensate for temperature hysteresis.

## Figures and Tables

**Table 1:** Training set.

Temperature (°C)	U (mV)										
	0 kN	10 kN	20 kN	30 kN	40 kN	50 kN	40 kN	30 kN	20 kN	10 kN	0 kN
0	0.011	0.6083	1.2067	1.8029	2.4024	2.9953	2.3969	1.8007	1.2012	0.6061	0.011
5	0.0044	0.6105	1.2177	1.8194	2.4189	3.025	2.4178	1.8139	1.2133	0.605	0.0044
10	−0.0055	0.6061	1.2122	1.8227	2.4299	3.0382	2.4266	1.8194	1.2045	0.5995	−0.0055
15	−0.0011	0.6171	1.2177	1.8271	2.4343	3.0415	2.4299	1.8216	1.2133	0.605	−0.0011
20	0	0.6149	1.2221	1.8348	2.4431	3.0525	2.4398	1.8282	1.2199	0.6083	0
25	−0.0121	0.6061	1.221	1.8348	2.4519	3.0624	2.4409	1.8293	1.2133	0.5984	−0.0121
30	−0.0132	0.6028	1.2166	1.8293	2.4442	3.0547	2.4387	1.8249	1.2122	0.5973	−0.0132
35	0.0055	0.6149	1.2221	1.8238	2.431	3.0382	2.4277	1.8216	1.2155	0.6083	0.0055
40	0.0165	0.6248	1.2254	1.8304	2.4376	3.036	2.4288	1.8227	1.221	0.6193	0.0165
45	0.0286	0.6336	1.2287	1.8326	2.4343	3.0327	2.4299	1.8271	1.232	0.6259	0.0286
50	0.0187	0.6237	1.2276	1.8304	2.4343	3.0338	2.4277	1.8238	1.2221	0.6193	0.0187
55	0.0099	0.6116	1.2133	1.8161	2.4167	3.0129	2.4101	1.8073	1.2089	0.6072	0.0099
60	0.0033	0.6083	1.2089	1.8106	2.4123	3.0096	2.4057	1.8062	1.2034	0.6017	0.0033

**Table 2:** Simulation results for different numbers of hidden layer neurons.

Number of Neurons	*e_r_*	*e_f_* %	MSE	RMSE
2	0.097	3.19	0.0021	0.046
3	0.042	1.38	0.00011	0.0106
4	0.046	1.71	0.00017	0.013
5	0.018	0.6	3.72 × 10^−5^	0.0061
6	0.0166	0.54	2.66 × 10^−5^	0.005
7	0.021	0.68	2.99 × 10^−5^	0.0055
8	0.023	0.76	4.56 × 10^−5^	0.0068
9	0.021	0.7	3.97 × 10^−5^	0.0063
10	0.016	0.52	2.64 × 10^−5^	0.0051

**Table 3:** Calibration data at full temperature.

		γ0 (ppm/°C)	γs (ppm/°C)	er	ef %	MSE	RMSE
	
Experiment		374.8	936.94	0.057	1.87	2.78 × 10^−4^	0.017
HBA-BP		342.64	478.8	0.084	2.74	0.0012	0.035
IHBA(no Levy)-BP		117.3	49.35	0.028	0.93	4.7 × 10^−5^	0.0069
IHBA(no g)-BP		102.99	101.45	0.0174	0.57	4.35 × 10^−5^	0.0066
IHBA-BP		35.09	45.75	0.0166	0.54	2.66 × 10^−5^	0.005
IMA-BP		177.06	246.62	0.019	0.62	3.87 × 10^−5^	0.006
PSO-BP		80.83	70.71	0.0178	0.58	3.13 × 10^−5^	0.0056
BP		47.51	115.19	0.018	0.6	4.02 × 10^−5^	0.0063
Polynomial Fitting		257.85	254.89	0.02	0.65	5.1 × 10^−5^	0.0071

**Table 4:** Test set.

Temperature (°C)	U (mV)					
	0 kN	10 kN	20 kN	30 kN	40 kN	50 kN
27	−0.0125	0.6048	1.2192	1.8326	2.4488	3.0593
37	0.0099	0.6189	1.2234	1.8264	2.4336	3.0373
47	0.0246	0.6296	1.2283	1.8317	2.4343	3.0331
57	0.0073	0.6103	1.2115	1.8139	2.4149	3.0116

**Table 5:** Compensation results after IHBA-BP.

Temperature (°C)	U (mV)					
	0 kN	10 kN	20 kN	30 kN	40 kN	50 kN
27	−0.0038	0.6076	1.217	1.8289	2.4552	3.05
37	−0.0009	0.6122	1.2233	1.8245	2.4418	3.0496
47	0.0022	0.6187	1.2327	1.8397	2.4508	3.0495
57	−0.0005	0.6119	1.2191	1.8345	2.4387	3.0485

## Data Availability

All data generated or analyzed during this study are included in this published article.
